# Safety Assessment of *Eubacterium limosum* El1405 and Its Protective Effect Against *Salmonella* Typhimurium Infection in Mice

**DOI:** 10.3390/nu18111738

**Published:** 2026-05-28

**Authors:** Yao Lu, Xiaoying Lin, Ruiting Lan, Ying Du, Xiaohui Zhou, Zheyu Yuan, Liyun Liu, Jianguo Xu

**Affiliations:** 1 National Key Laboratory of Intelligent Tracking and Forecasting for Infectious Diseases, National Institute for Communicable Disease Control and Prevention, Chinese Center for Disease Control and Prevention, Beijing 102206, China; willongluyao@163.com (Y.L.);; 2 School of Public Health, Nanjing Medical University, Nanjing 211166, China; 3 School of Biotechnology and Biomolecular Sciences, University of New South Wales, Sydney, NSW 2052, Australia; 4 Hebei Key Laboratory of Intractable Pathogens, Shijiazhuang Center for Disease Control and Prevention, Shijiazhuang 050011, China; 5 Research Center for Reverse Microbial Etiology, Workstation of Academician, Shanxi Medical University, Taiyuan 030001, China

**Keywords:** *Eubacterium limosum*, safety assessment, *Salmonella* Typhimurium infection, probiotics

## Abstract

**Background/Objectives:** *Eubacterium limosum* El1405 is a novel probiotic candidate strain that has been shown to exert prominent anti-inflammatory and anti-tumor bioactivities, with potential antibacterial activity against pathogenic bacteria. This study aimed to systematically evaluate the safety of *E. limosum* El1405 and its probiotic functions and protective effects against pathogenic bacterial infection. **Methods:** The safety of *E. limosum* El1405 was assessed through in vitro assays of hemolytic and gelatinase activities and a 28-day subchronic oral toxicity mouse model. In the mouse model, three different doses (low, medium, and high) of *E. limosum* El1405 were tested, and physiological status, visceral histopathology, hematological profiles, serum biochemistry, and cytokines were measured. The antibacterial activity of the strain against pathogenic bacteria was determined in vitro. A *Salmonella* Typhimurium -infected mouse model was used to assess its potential to protect against infection. **Results:** In vitro safety assays confirmed that *E. limosum* El1405 possessed no hemolytic or gelatinase activity. In the 28-day subchronic oral toxicity test, low, medium, and high doses of El1405 caused no significant alterations in mouse body weight, visceral index, organ histopathology, hematological parameters, serum biochemistry, or cytokine levels. The strain exhibited antibacterial activity against *S.* Typhimurium in vitro. In *S.* Typhimurium-infected mice, El1405 intervention effectively mitigated *S.* Typhimurium-induced damage, reduced visceral bacterial loads, decreased pro-inflammatory cytokines (TNF-α, IL-6, IL-1β) in the ileum and serum, and elevated the anti-inflammatory cytokine IL-10. **Conclusions:** *E. limosum* El1405 displays a favorable safety profile and promising probiotic effects, including antibacterial capacity and anti-inflammatory protective effects against *S.* Typhimurium infection, supporting further exploration and development of *E. limosum* El1405 as a novel functional probiotic strain for clinical and health applications.

## 1. Introduction

Probiotics are live microorganisms that are capable of conferring health benefits on the host when administered at adequate doses. They exert beneficial effects through various mechanisms, including the reduction of intestinal pH, inhibition of pathogenic microbial colonization and invasion, and modulation of the host immune response [1]. Numerous studies have demonstrated that probiotics of varying species or strains can effectively alleviate different types of diseases [2]. Currently, novel or specific categories of candidate probiotic strains are being identified. Before incorporating new strains into products, it is essential to conduct a comprehensive evaluation of their efficacy and safety to ascertain whether these strains possess food-grade safety attributes comparable to those of traditional probiotic strains [3]. Food-grade safety attributes refer to the inherent safety characteristics of the strain, including being classified as Generally Recognized As Safe (GRAS) by the US FDA or included in the Qualified Presumption of Safety (QPS) list by the European EFSA, absence of functional virulence factors and transferable antimicrobial resistance genes, as well as non-hemolytic and non-pathogenic phenotypes in vitro and in vivo [4]. Notably, the safety of probiotics depends on dosage and duration, as well as the mode and frequency of administration, and their intended use and the health of the user. Based on the FAO/WHO official definition, probiotics are defined as live microorganisms that must confer a health benefit on the host when administered in adequate amounts. This stringent requirement necessitates that all strains be systematically and thoroughly studied before their application in humans or animals [4].

*Eubacterium* is a part of the core human gut microbiome. Certain species within the genus *Eubacterium*, such as *Eubacterium limosum*, *Eubacterium callanderi*, and *Eubacterium rectale*, are recognized for their capacity to produce butyrate [5,6]. Butyrate plays multiple roles in gut health, including maintaining energy homeostasis, regulating colonic motility, modulating immune responses, and suppressing intestinal inflammation [7]. Specific butyrate-producing strains of *Eubacterium* may ultimately be regarded as beneficial to human health, similar to the well-known strains of *Lactobacillus* and *Bifidobacterium* [8]. In our previous study, the *E. limosum* strain El1405 was shown to produce metabolites, including indole-3-acetic acid and indole-3-lactic acid, which alleviate inflammatory bowel disease and inhibit colorectal cancer [9,10]. However, before advancing toward further preclinical evaluation and eventual clinical translation, a comprehensive and systematic assessment of its biosafety profile is indispensable to ensure its reliability and safety for in vivo application. Furthermore, our previous study revealed that *E. limosum* El1405 could reduce the abundance of various opportunistic pathogens, including *Staphylococcus* species [9,10], suggesting its potential to protect against pathogenic bacterial infections.

Nontyphoidal *Salmonella* gastroenteritis, caused by *Salmonella,* including *Salmonella enterica* subsp. *enterica* serovars Typhimurium and Enteritidis, ranks among the most prevalent foodborne diseases worldwide. The estimated global burden of non-typhoidal *Salmonella* gastroenteritis exceeds 93.8 million cases annually, indicating a significant impact on public health worldwide [11]. *S*. Typhimurium is a prominent serovar of *Salmonella* found worldwide, known for causing outbreaks of self-limiting gastroenteritis that are largely associated with the industrialization of food production. This pathogen exhibits a broad host range and remarkable metabolic versatility [12]. *Salmonella* infection may require treatment with antibiotics. However, the widespread overuse of antibiotics in farm animals led to the emergence of antibiotic-resistant *Salmonella*, which is becoming an increasing concern [13]. Given that *Salmonella* is an important foodborne pathogen posing a significant public health threat, this study further focused on investigating the interventional and protective effects of *E. limosum* El1405 against *Salmonella* infection.

The rising prevalence of antibiotic-resistant pathogens underscores the urgent need to explore novel strategies for the prevention and treatment of intestinal infections. In this context, probiotics have emerged as a potential avenue for the prevention and treatment of *Salmonella* infections, owing to their safety, lack of drug resistance, and ability to regulate intestinal microecology [14]. A substantial number of in vitro experiments and animal model studies confirmed that specific probiotic strains can exert anti-*Salmonella* effects through various mechanisms [15]. The *Escherichia coli* strain Nissle 1917 effectively mitigates intestinal colonization by *S*. Typhimurium by competing for the limited bioavailable iron [16]. *Lactobacillus casei* CRL 431 has been shown to alleviate the severity of *S*. Typhimurium infection [17]. Studies have shown that *Lacticaseibacillus rhamnosus* GG can promote intestinal ILC3 activation via the TLR2 receptor, which in turn inhibits *S*. Typhimurium infection in murine models [18]. Probiotics and their bioactive metabolites have emerged as promising alternative therapeutic agents that not only reduce reliance on antibiotics but also enhance the efficacy of conventional pharmacotherapies. However, the anti-*Salmonella* potential of many probiotic strains remains uncharacterized, as functional effects of probiotics could be strain-specific [19].

In this study, we systematically characterize the biosafety of *E. limosum* El1405. We assessed the general and subacute toxic effects of El1405 in male and female BALB/c mice in accordance with the Organisation for Economic Cooperation and Development (OECD) guidelines [9,20]. We further used a mouse model of *S*. Typhimurium infection to evaluate the protective effect of the candidate probiotic *E. limosum* El1405 against *Salmonella* infection.

## 2. Materials and Methods

### 2.1. Bacterial Culture Conditions

El1405 was previously isolated from healthy human feces [9] and was obtained from the China Microbial Culture General Collection Center (CGMCC) under the preservation number CGMCC NO. 31231 [9]. El1405 was anaerobically cultured on reinforced clostridial medium (RCM) plates at 37 °C for 48 h. The culture method for the El1405 culture supernatant (El1405CS) is based on established protocols from previous studies [9]. El1405CS was filtered through a 0.22 μm Millipore filter membrane. Subsequently, El1405CS was concentrated to a 10× concentration using an Alpha 2-4 LSC basic laboratory lyophilizer (CHRIST, Osterode am Harz, Germany). Finally, the concentrated supernatant was filtered once more through a 0.22 μm filter membrane to obtain the sterile 10× El1405CS.

*S*. Typhimurium strain SL1344, *L. rhamnosus* GG, *L. monocytogenes* EGDe, *S. aureus* ATCC 25923, and *E. coli* EDL933 were cultured at 37 °C in Luria–Bertani (LB) broth for 12 h. After revival from stored stock, cultures were subcultured daily to maintain freshness for experiments at passages 3–5. Strains were re-cultured from the original stock to maintain their biological stability.

### 2.2. Antibiotic Susceptibility

According to the Clinical and Laboratory Standards Institute (CLSI) M100 standard, the following antibiotics are recommended for testing anaerobic bacteria: penicillin, ampicillin, imipenem, meropenem, amoxicillin, ceftriaxone sodium, clindamycin, moxifloxacin, metronidazole, and tetracycline. These antibiotics were employed to evaluate the antibiotic susceptibility of *E. limosum* El1405. In this study, *Bacteroides fragilis* ATCC 25285 was utilized as a quality control strain (QC) to determine its minimum inhibitory concentration (MIC) value through the antibiotic concentration gradient method, specifically the E-test method.

### 2.3. Whole-Genome Sequencing and Analysis

Genome sequencing, assembly, and gene prediction of strain El1405 were performed as previously reported by our research group [9]. Whole-genome sequencing data of strain El1405, as previously established, were used in this study for the systematic annotation and screening of virulence and antimicrobial resistance genes.

The Resistance Gene Identifier (RGI, v6.0.5) was used to annotate antimicrobial resistance (AMR) genes against the curated Comprehensive Antibiotic Resistance Database (CARD, v4.0.1). Open Reading Frame (ORF) prediction was performed using software Prodigal (v2.6.3), and homolog detection was conducted with software DIAMOND (v2.1.8). Only perfect and strict matches defined by CARD curated bitscore cut-offs were retained for the identification of resistance genes [21].

### 2.4. Gelatinase Activity

To prepare a 3% gelatin-Brain–Heart Infusion Broth (BHI medium), adjust the OD_600_ of El1405 to 0.6. Next, instill 10 µL of the sample onto the medium and incubate at 37 °C under anaerobic conditions for 48 h, until colony formation is observed. Following incubation, pour saturated ammonium sulfate onto the plate and observe for halo formation. *S. aureus* ATCC 25923 was utilized as a positive control, and *L. rhamnosus* GG was utilized as a negative control.

### 2.5. Inhibition of the Pathogen

The concentration of El1405 was adjusted to 1 × 10^8^ CFU/mL using PBS. Subsequently, 10 μL of the suspension was inoculated onto RCM agar plates, followed by anaerobic incubation at 37 °C for 24 h until colonies formed. The concentration of the pathogens to be tested was adjusted to 1 × 10^9^ CFU/mL. Pathogen suspension was mixed with 0.6% agar LB medium at a volume ratio of 0.1% (*v*/*v*), and applied over the RCM plate containing the colonies. After drying, the plates were incubated at 37 °C for 4 to 12 h.

The Oxford cup assay was used to assess the antimicrobial activity of the 10× concentrated culture supernatant produced by El1405. Prepare 1.5% agar-LB medium using oxford cups. The concentration of the pathogenic bacteria to be tested was adjusted to 1 × 10^9^ CFU/mL, and then the LB plate was uniformly coated with a cotton swab. Subsequently, 200 μL of 10× El1405CS was added to each well, and the plates were cultured at 37 °C for 4–12 h. RCM medium served as the negative control. The diameter of each inhibition zone was measured with a vernier caliper.

### 2.6. Animal Experiments

#### 2.6.1. Assessment of the Safety

Specific-pathogen-free (SPF) female (6–8 weeks old, 18–20 g) and male (6–8 weeks old, 21–23 g) BALB/c mice were purchased from Vital River Lab Animal Technology Co., Ltd. (Beijing, China). The mice were housed under specific pathogen-free conditions at a temperature of 23 ± 2 °C and a relative humidity of 55 ± 5%, and subjected to a 12 h light–dark cycle. After one week of adaptive feeding, mice were randomly divided into eight experimental groups.

Each treatment group included 5 male and 5 female mice. The sample size was determined based on statistical power requirements to ensure reliable and reproducible experimental results. All female and male mice were randomly assigned to four experimental groups: the negative control (NC) group (0.2 mL PBS daily), the high-dose group (1 × 10^9^ CFU El1405/0.2 mL daily), the medium-dose group (5 × 10^8^ CFU El1405/0.2 mL daily), and the low-dose group (1 × 10^8^ CFU El1405/0.2 mL daily). Eight groups of mice were subjected to oral toxicity tests for a duration of 28 days. The weight of the mice was measured daily, while food and water intake were assessed every week. Experimental mice were included strictly according to unified criteria involving health status, age, and body weight. No animals, experimental units, or data were excluded throughout the study. All data analyses were performed using standardized procedures to ensure unbiased results.

After a 12 h fasting period on day 29, all mice were euthanized. Animals were first anesthetized via gradual CO_2_ inhalation with a 30% chamber air replacement rate, and cervical dislocation was subsequently applied to confirm complete death. The whole blood and serum samples were collected. Whole blood was utilized for routine blood testing, while serum was analyzed for biochemical and immune factors. The heart, liver, spleen, lungs, and kidneys were weighed, and their relative weights were calculated. The tissues were stained with HE to assess any pathological changes.

#### 2.6.2. S. Typhimurium Infection in Mice

A total of 24 female SPF-grade BALB/c mice (5–6 weeks old, 16–18 g) were randomly allocated to three experimental groups: the negative control (NC) group (0.2 mL PBS daily), the PBS group (0.2 mL PBS daily), and the El1405 group (1 × 10^8^ CFU El1405/0.2 mL daily). On day 0, each mouse in the PBS and El1405 groups was gavaged with 1 × 10^8^ CFU of *S.* Typhimurium SL1344. From day −7 to day 6, the El1405 group received daily oral gavage of El1405 at 1 × 10^8^ CFU in 0.2 mL PBS, and the PBS group was given an equal volume (0.2 mL) of PBS. The weight of the mice was measured daily, and they were sacrificed on day 6. Subsequently, serum, liver, and spleen samples were collected. A total of 0.1 g of liver and spleen tissue was quantitatively weighed, and then 1 mL of PBS, along with 3 mm steel beads, were added to create a tissue homogenate. A *Salmonella* selective medium containing streptomycin (50 μg/mL) was prepared for counting the colonies of *Salmonella* bacteria present in the liver and spleen of the mice.

### 2.7. Histological Analysis

Tissue specimens from the heart, liver, spleen, lungs, kidneys, and distal colon were initially embedded in 4% paraformaldehyde for histological analysis. Cross-sections were then stained with HE. The method was determined according to previously published literature [10]. All tissues are examined by a pathologist for comprehensive histopathology.

### 2.8. Hematology and Biochemical Testing

Whole blood (100 µL) was collected into EDTA anticoagulant tubes for complete blood count (CBC) analysis. The leftover blood sample was processed to isolate serum via centrifugation at 3000× *g* for 15 min under 4 °C conditions. A Mindray BC-2800Vet hematology analyzer (Mindray, Shenzhen, China) was adopted to detect CBC parameters, and serum biochemical parameters were assayed using a Hitachi 3500 automatic biochemistry analyzer (Hitachi, Tokyo, Japan).

### 2.9. Enzyme-Linked Immunosorbent Assay (ELISA)

Cytokine levels in the serum and ileum, including TNF-α, IL-4, IL-12, IL-6, IL-10, IL-1β, and IFN-γ, were assessed using ELISA kits (Dogesce, Beijing, China). Serum samples were diluted twofold before ELISA testing. For the colon, 0.1 g of ileum tissue was weighed, and 1 mL of PBS was added for homogenization. Following this, ELISA detection was conducted.

### 2.10. Statistical Analysis

Statistical analysis was performed using GraphPad Prism 9.0. The data were expressed as the mean ± Standard Error of the Mean (SEM). Differences between two groups were tested using Student’s *t*-test. Differences among multiple groups were evaluated using one-way ANOVA, followed by either Dunnett’s or Tukey’s multiple comparison test. Body weight changes in mice were evaluated using two-way ANOVA. *p*-value < 0.05 was considered statistically significant. ns indicates no significant difference. * *p* < 0.05, ** *p* < 0.01, *** *p* < 0.001, and **** *p* < 0.0001.

## 3. Results

### 3.1. In Vitro Safety, Antibiotic Susceptibility, Antibacterial Activity, and Genomic Safety Evaluation of Strain El1405

*E. limosum* El1405 is a Gram-positive bacterium that cannot produce endotoxins and demonstrates a certain level of safety (Figure S1A). No hemolytic or gelatinase activity was detected in El1405 (Figure S1B,C).

El1405 showed sensitivity to all antibiotics tested except penicillin, including ampicillin, amoxicillin, imipenem, meropenem, clindamycin, ceftriaxone, moxifloxacin, tetracycline, and metronidazole (Table S1).

Genome sequencing of strain El1405 revealed the presence of one chromosome and one plasmid, and genomic analysis was further performed to screen for AMR genes. Four AMR genes were identified in the chromosome of strain El1405 via RGI analysis against the CARD database: three glycopeptide resistance-related genes, *vanT* (35.97% identity, 56.04% coverage), *vanY* (32.27% identity, 97.76% coverage), and *vanH* (37.74% identity, 93.71% coverage), and one tetracycline resistance gene, *tet(Q)* (31.72% identity, 99.39% coverage). All genes showed low amino acid identity (<40%) with known reference sequences, indicating limited similarity to characterized AMR determinants.

El1405 showed significant antibacterial activity against four pathogens tested, with the strongest effect against *Staphylococcus aureus* ATCC 25923, followed by *S*. Typhimurium SL1344 and *E. coli* EDL933, and the weakest against *Listeria monocytogenes* EGDe. While the culture supernatant of El1405 retained activity against the other strains except *L. monocytogenes* EGDe (Table S2).

### 3.2. The Repeated Dose 28-Day Oral Toxicity Test

**Figure 1:**
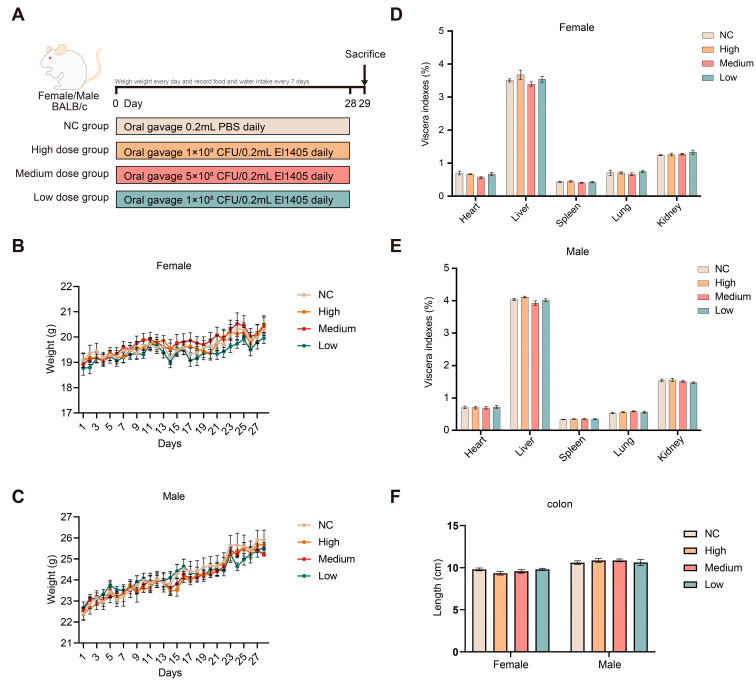
The effects of *E. limosum* El1405 on the growth, development, and visceral indexes of mice. (**A**) Schematic of the experimental procedure; (**B**) body weight change curves of female mice; (**C**) body weight change curves of male mice; (**D**) viscera indexes of female mice; (**E**) viscera indexes of male mice; (**F**) the colon length of mice. NC: negative control. High: gavage with high-dose El1405. Medium: gavage with medium-dose El1405. Low: gavage with low-dose El1405. Number of mice per group = 5 for each gender. Statistical tests were conducted in (**D**–**F**) among the groups. No significant differences were found (*p* > 0.05).

The subacute oral toxicity of El1405 was assessed in BALB/c mice through a 28-day repeated-dose study. The experimental approach is illustrated in Figure 1A. Throughout the experiment, no mortality or morbidity was observed in any mice in the treatment group throughout the study period. Compared with the control group, mice in all experimental groups showed no significant difference in food and water intake as well as body weight (Table S3, Figure 1B,C). After the 28-day experiment, the hearts, livers, spleens, lungs, and kidneys of the mice from each group were collected and weighed, and the visceral index was computed. Compared with the control group, no significant differences were observed in the visceral index of mice among all experimental groups (Figure 1D,E). Furthermore, under high, medium, and low doses of El1405, there were no significant changes in colon length among the experimental groups relative to the control group (Figure 1F). These findings indicated that El1405 had no obvious impact on the growth and development of mice at the tested doses, indicating that El1405 possesses a favorable safety profile.

**Figure 2:**
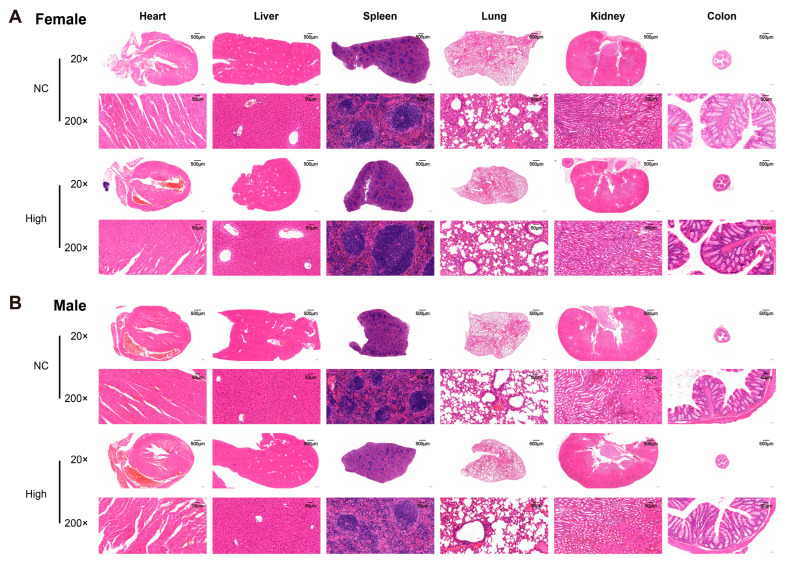
The effects of *E. limosum* El1405 on organs in the subacute toxicity study. (**A**) Representative image of HE staining of female mouse organs. Scale bar = 50 and 500 μm. (**B**) Representative image of HE staining of male mouse organs. Scale bar = 50 and 500 μm. NC: negative control. High: gavage with high-dose El1405. Number of mice per group = 5 for each gender.

To further assess the potential damage to the primary organs of mice subjected to El1405 intervention, the heart, liver, spleen, lung, kidney, and colon tissues were stained with hematoxylin–eosin (HE) to assess any pathological changes. Representative images are illustrated in Figure 2. Histopathological analysis revealed no significant histological differences compared with the control group, such as hyperemia, degeneration, necrosis, hyperplasia, or inflammation, in the major organs of mice treated with a high dose of El1405. These results further demonstrate that El1405 does not induce significant pathological changes in the primary organs of mice, suggesting that long-term intervention with El1405 as a probiotic can safeguard the health of mouse organs and confirm the safety of El1405.

**Figure 3:**
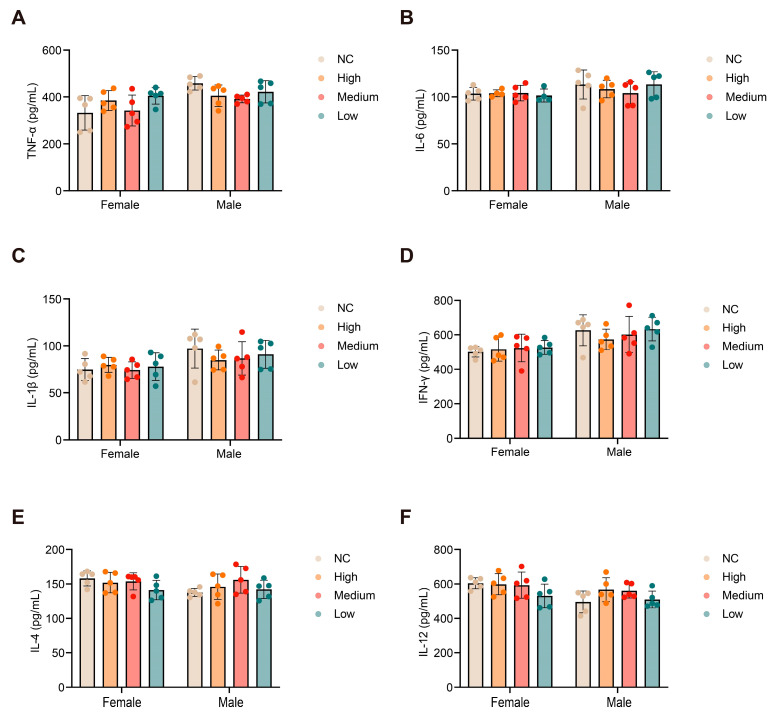
The effects of *E. limosum* El1405 on serum cytokines in mice. (**A**–**F**) Levels of cytokines, TNF-α, IL-6, IL-1β, INF-γ, IL-4, and IL-12, in the serum of mice. NC: negative control. High: gavage with high-dose El1405. Medium: gavage with medium-dose El1405. Low: gavage with low-dose El1405. Number of mice per group = 5 for each gender. Statistical tests were conducted among the groups and no significant differences were found (*p* > 0.05).

Additionally, the effects of El1405 on mouse health were further assessed by measuring 17 hematological parameters and eight serum biochemical indicators, and no significant differences were observed among the treatment and control groups (Tables S4 and S5). Cytokines, TNF-α, IL-6, IL-1β, INF-γ, IL-4, and IL-12 in the serum following different doses of strain El1405 intervention were measured using ELISA. Again, no significant differences were found in both male and female mice among the treatment and control groups (Figure 3A–F). These results indicate that strain El1405 has no adverse impact on the hematological parameters of mice, supporting its characterization as safe.

### 3.3. El1405 Relieves S. Typhimurium Infection in Mice

**Figure 4:**
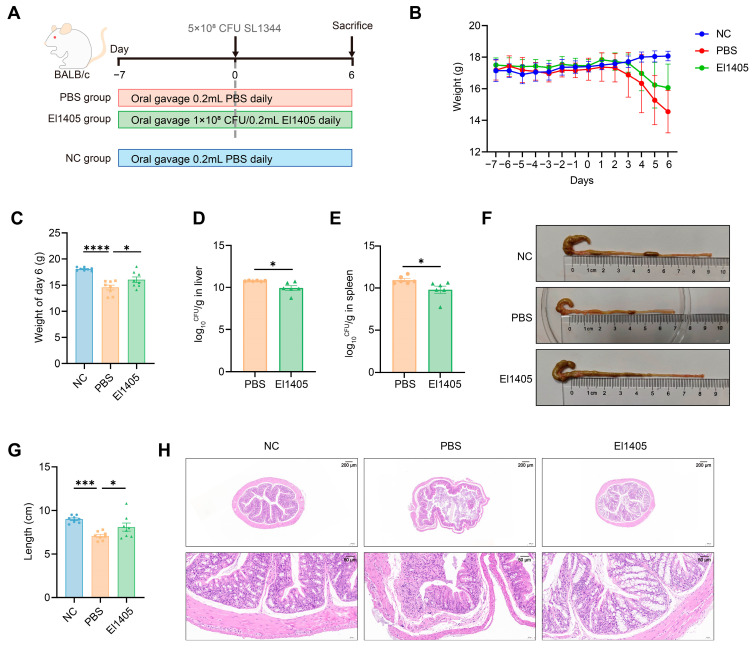
*E. limosum* El1405 relieves *S.* Typhimurium infection in mice. (**A**) Schematic of the experimental procedure; (**B**) body weight change curves of mice; (**C**) weight on Day 6; (**D**) the colonies of *Salmonella* bacteria present in the liver of the mice; (**E**) the colonies of *Salmonella* bacteria present in the spleen of the mice; (**F**) representative images of the colons of mice from different treatment groups; (**G**) the colon length of mice; (**H**) representative images of colon tissue sections stained with HE. Scale bar = 50 and 200 μm. NC: negative control. PBS: gavage with PBS. El1405: gavage with El1405. Number of mice per group = 8 for each group. Data are presented as mean ± SEM. * *p* < 0.05, *** *p* < 0.001, and **** *p* < 0.0001.

To evaluate the effect of *E. limosum* on the prevention of bacterial infection, we utilized a mouse model of *S*. Typhimurium infection. Female BALB/c mice were orally gavaged with 1 × 10^8^ CFU/0.2 mL of El1405 or 0.2 mL Phosphate-Buffered Saline (PBS) from day −7 to day 6. On day 0, the mice in both the PBS and El1405 groups were orally gavaged with 5 × 10^8^ CFU of *S*. Typhimurium SL1344 (Figure 4A). Following infection with SL1344, both the PBS and El1405 groups exhibited a trend of weight loss. However, the El1405 group demonstrated a reduced rate of weight loss compared to the PBS group (Figure 4B). On day 6, body weight in the PBS group was significantly decreased relative to the NC group, while the El1405 group showed a markedly higher body weight than the PBS group (Figure 4C). Colony counts from the liver and spleen of mice in each group indicated that El1405 significantly reduced organ bacteria load in *S*. Typhimurium-infected mice (Figure 4D,E). Compared to the NC group, the PBS group exhibited a significant reduction in colon length, while the El1405 grouped had reduced colonic shortening when compared to the PBS group (Figure 4F,G).

**Figure 5:**
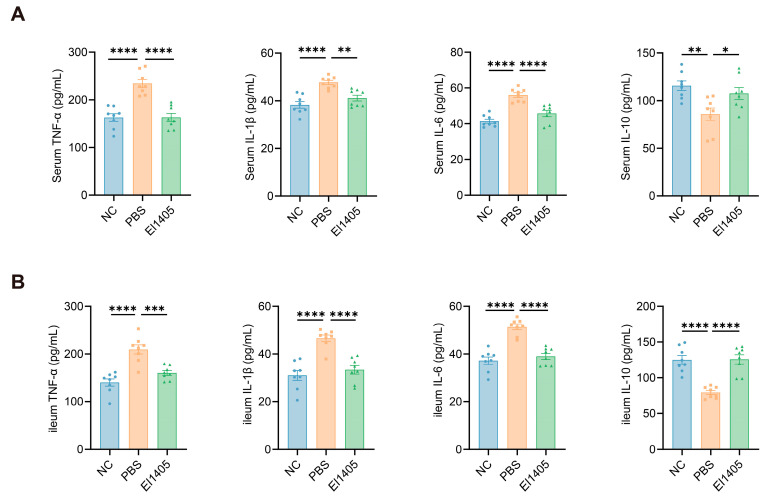
The effects of *E. limosum* El1405 on serum cytokines in *S. Typhimurium*-infected mice. (**A**) Levels of cytokines, TNF-α, IL-1β, IL-6, and IL-10, in the serum of mice. (**B**) Levels of cytokines, TNF-α, IL-1β, IL-6, and IL-10, in the ileum of mice. NC: negative control. PBS: gavage with PBS. El1405: gavage with El1405. Number of mice per group = 8 for each group. Data are presented as mean ± SEM. * *p* < 0.05, ** *p* < 0.01, *** *p* < 0.001, and **** *p* < 0.0001.

HE staining and histological analysis indicated that *S*. Typhimurium induced significant ulceration of the colon, resulting in the loss of mucosal epithelium and intestinal glandular structure, accompanied by connective tissue hyperplasia and lymphocyte infiltration. In contrast, the El1405 group had reduced colon inflammation in mice infected with *S*. Typhimurium (Figure 4H). Furthermore, compared to the NC group, the PBS group had significantly elevated serum and ileum pro-inflammatory factors, including TNF-α, IL-1β, and IL-6, and reduced anti-inflammatory factors such as IL-10. Compared to the PBS group, the El1405 group significantly decreased the levels of pro-inflammatory factors in both serum and ileum, and increased the levels of anti-inflammatory factors in these tissues (Figure 5A,B). These results suggest that El1405 can alleviate *S*. Typhimurium infection and mitigate the inflammatory response in mice.

## 4. Discussion

Ensuring the safety of probiotics is crucial for human consumption and the safety profile is often strain-specific [22]. To support the development of *E. limosum* El1405 as a probiotic candidate for adjuvant therapy, it is essential to verify its safety. In this study, we comprehensively evaluated the safety profile of El1405 and found no undesirable properties from both in vitro and in vivo testing.

The beneficial effects of probiotics in regulating intestinal microecology and alleviating inflammatory bowel disease have been well established [23]. However, the increasing use of probiotics in clinical and food applications has raised concerns regarding their potential safety risks [24]. The safety assessment of probiotics must adhere strictly to standardized guidelines. The OECD toxicity testing method has emerged as a critical reference for evaluating the safety of food-grade microorganisms due to its scientific rigor and normative characteristics [20]. According to OECD No. 407 [20], the repeated dose 28-day oral toxicity test is classified as a subacute toxicity test. This test is primarily utilized to assess the potential toxic effects of prolonged oral exposure to substances such as drugs and probiotics on experimental animals. It serves to provide essential safety data that supports subsequent long-term chronic toxicity tests or clinical studies [25]. In this study, a 28-day repeated administration assay was performed to assess the toxicity of strain El1405, to determine its dose–response relationship, and identify any long-term adverse effects. The results showed that daily oral administration of a high dose of El1405 (1 × 10^9^ CFU/day equivalent to 5 × 10^10^ CFU/kg) for 28 days was safe and well tolerated in both male and female mice. It represents 3500 times the empirical dose level of oral probiotics in humans (equivalent to 1 × 10^9^ CFU/day or 1.43 × 10^7^ CFU/day/kg in a 70 kg individual) [26]. The El1405-treated groups (containing low-dose group, medium-dose group, and high-dose group) showed no abnormal changes, no significant organ damage, or abnormal physiological indicators compared with the control group in the subacute experiments, including the body weights, feed consumption, visceral index, histopathology, hematological parameters, serum biochemical indicators, and serum cytokines. It is noteworthy that El1405, isolated from the gut of healthy individuals, may enhance its intestinal adaptability and mitigate potential risks of immune rejection or metabolic disorders associated with exogenous strains, thus demonstrating its safety advantages.

Hematological parameters reflect the physiological status of peripheral blood and serve as fundamental indicators of overall systemic health [27]. Concurrently, the assessment of liver function-related serum biochemical markers constitutes a key diagnostic approach for evaluating metabolic toxicity [28]. All hematological and serum biochemical indicators in the El1405 treatment groups remained within the normal physiological ranges and were comparable to those of the NC group. These findings indicate that El1405 did not exert any adverse effects on liver, kidney, or cardiac function, nor did it impair systemic metabolic homeostasis in healthy mice.

Cytokine expression levels are indicative of the body’s inflammatory response, immune homeostasis, and tissue damage status [29]. In the present study, the analysis of cytokine profiles (TNF-α, IL-1β, IL-6, IFN-γ, IL-4, IL-12) demonstrated no significant differences between the El1405-treated group and the control group, thereby confirming that the El1405 strain does not induce systemic immune stimulation. Further studies on the long-term exposure effects of El1405 (longer than 90 days) and its potential effects on people with compromised immune systems are needed to ensure that these special populations do not face additional health risks.

Probiotic strains can possess such resistance genes on mobile genetic elements (MGEs) [24], which becomes a concern as these genes may be transferred to gut commensals or opportunistic pathogens. Consequently, the absence of acquired antimicrobial resistance is one of the primary safety criteria for screening candidate probiotic strains [30]. In this study, no antimicrobial resistance genes were detected in the plasmid sequences of strain El1405, confirming that all identified AMR genes are chromosomally encoded rather than plasmid-borne, with no risk of plasmid-mediated resistance transfer. Genomic analysis revealed that only four AMR genes were present on the chromosome of strain El1405, with no typical genotypes matching the tested antibiotics. Three glycopeptide resistance-related genes, *vanT*, *vanY*, *vanH*, and one tetracycline resistance gene *tet(Q)* were identified, all sharing amino acid homology less than 40% with reference sequences in the CARD database. Such low sequence similarity indicated that these genes were phylogenetically distant from well-characterized resistance determinants, which partly explained the susceptible phenotype of strain El1405 to tetracycline, although any resistance to glycopeptide antibiotics is unknown. Furthermore, the strain exhibited phenotypic resistance to penicillin, whereas no corresponding resistance gene was detected, which may be attributed to adaptive resistance induced by the human intestinal microenvironment. Overall, the genomic resistance profile confirmed the low antibiotic resistance risk of this strain at the genetic level.

El1405 inhibited all four pathogens tested in vitro (*L. monocytogenes* EGDe, *S. aureus*, *S*. Typhimurium, and *E. coli*). In contrast, its culture supernatant inhibited *S. aureus*, *S*. Typhimurium, and *E. coli*, but was ineffective against *L. monocytogenes* EGDe, indicating that El1405 exerts its antibacterial effect through different mechanisms. Based on these in vitro findings, we then used a mouse model to investigate whether El1405 confers protection against *S*. Typhimurium infection in vivo. We found that *E. limosum* El1405 can alleviate the pathological symptoms associated with *S*. Typhimurium infection in mice. Specifically, it reduces infection-induced weight loss, inhibits the increase of *S*. Typhimurium bacterial load in the liver and spleen, and improves colon shortening. The ileum is the organ most affected by the initial invasion of *S*. Typhimurium, exhibiting progressive injury, increased translocation of pathogenic cells into peripheral blood circulation [31]. Given that serum immune factors provide a more accurate reflection of the overall changes in the systemic immune response, we simultaneously detected the expression levels of immune factors in both mouse serum and ileum tissue to comprehensively assess the local and systemic immune status of the host. We found that El1405 significantly decreased the levels of pro-inflammatory factors (TNF-α, IL-6, IL-1β) in the ileum and serum of mice, while increasing the levels of the anti-inflammatory factor IL-10. Similarly, the *L. rhamnosus* strain HN001 has been demonstrated to reduce organ pathogen loads and enhance leukocyte phagocytic activity in mice infected with *Salmonella*, indicating that this strain exerts immunoenhancing effects and provides protection against enteric bacterial infection [32]. Pretreatment with *L. paracasei* ST11-fermented milk protected BALB/c mice against *S*. Typhimurium infection by suppressing the mRNA expression of ileal pro-inflammatory cytokines, including IFN-γ, IL-6, TNF-α, and IL-17. This intervention also reduced pathogen translocation and helped preserve the intestinal microbiota [33]. Probiotics have provided novel insights for designing alternative or adjuvant interventions to manage *Salmonella* infection, especially considering the growing challenge posed by antibiotic resistance. Further exploration of the synergy between El1405 and prebiotics or related drugs may confirm whether combined use strengthens anti-infection effects. Although in vitro antibacterial activities of strain El1405 were observed, the specific molecular mechanisms of these activities remain unclear and require further study. Overall, while our study confirms the anti-*Salmonella* infection capacity of El1405, more in-depth studies are necessary to fully establish its clinical application value.

## 5. Conclusions

This study has confirmed that *E. limosum* El1405 is safe with probiotic potential. Specifically, this strain is sensitive to nine of the 10 antibiotics tested, lacks virulence-related activities such as hemolysis and gelatinase production, and can inhibit common pathogenic bacteria in vitro. When administered at a dose of 5 × 10^10^ CFU/kg (equivalent to 1 × 10^9^ CFU/day) for 28 consecutive days, El1405 did not cause any alterations to hematological parameters or serum biochemical indices. Moreover, El1405 exerts a significant alleviating effect on *Salmonella*-infected mice: it can effectively suppress the elevation of pathogenic bacterial loads in the liver and spleen, ameliorate pathological changes, including colon shortening, and regulate immune responses by reducing the levels of pro-inflammatory cytokines (TNF-α, IL-6, and IL-1β) in the ileum and serum and increasing the level of the anti-inflammatory cytokine IL-10. In summary, El1405 is a probiotic strain with a favorable safety profile and considerable application potential.

## Data Availability

The whole-genome sequence of strain El1405 has been deposited in the GenBank database under the accession number PRJNA1170505. The original contributions presented in this study are included in the article/Supplementary Materials. Further inquiries can be directed to the corresponding authors.

## Supplementary Materials

The following supporting information can be downloaded at: https://www.mdpi.com/article/10.3390/nu18111738/s1, Figure S1: The results of *E. limosum* El1405 in vitro; Table S1: Antibiotic susceptibility of El1405; Table S2: The zone of inhibition diameter of El1405 live bacteria and supernatants; Table S3: Food and water intake of the negative control (NC) group and the experimental group; Table S4: Hematological parameters in mice of the negative control group (NC) and the experimental group; Table S5: Serum biochemical indicators of the negative control group (NC) and the experimental group.

## Abbreviations

The following abbreviations are used in this manuscript:

RCM	Reinforced clostridial medium
LB	Luria–Bertani broth
BHI	Brain–Heart Infusion Broth
PBS	Phosphate-buffered saline
CGMCC	China General Microbiological Culture Collection Center
ATCC	American Type Strain Preservation Center
OECD	Organisation for Economic Co-operation and Development
CLSI	Clinical and Laboratory Standards Institute
QC	quality control
MIC	minimum inhibitory concentration
HE	hematoxylin–eosin
CBC	complete blood count
