# Supplementary material for: Safety Assessment of Eubacterium limosum El1405 and Its Protective Effect Against Salmonella Typhimurium Infection in Mice
Source: Nutrients. 2026 May 28;18(11):1738. doi: 10.3390/nu18111738 (PMC13259198; doi:10.3390/nu18111738)
Supplement: Supplementary file 1 [file nutrients-18-01738-s001.zip › nutrients-4303260-supplementary.pdf]

Supplementary information for

Safety Assessment of *Eubacterium limosum* El1405 and Its Protective Effect Against *Salmonella* Typhimurium Infection in Mice

Yao Lu [1], Xiaoying Lin [1,2], Ruiting Lan [3], Ying Du [1,2], Xiaohui Zhou [1], Zheyu Yuan [1], Liyun Liu [1,4,*] and Jianguo Xu [1,2,5,*]

This PDF includes:

(1) Figures S1

(2) Supplementary Tables S1–S5

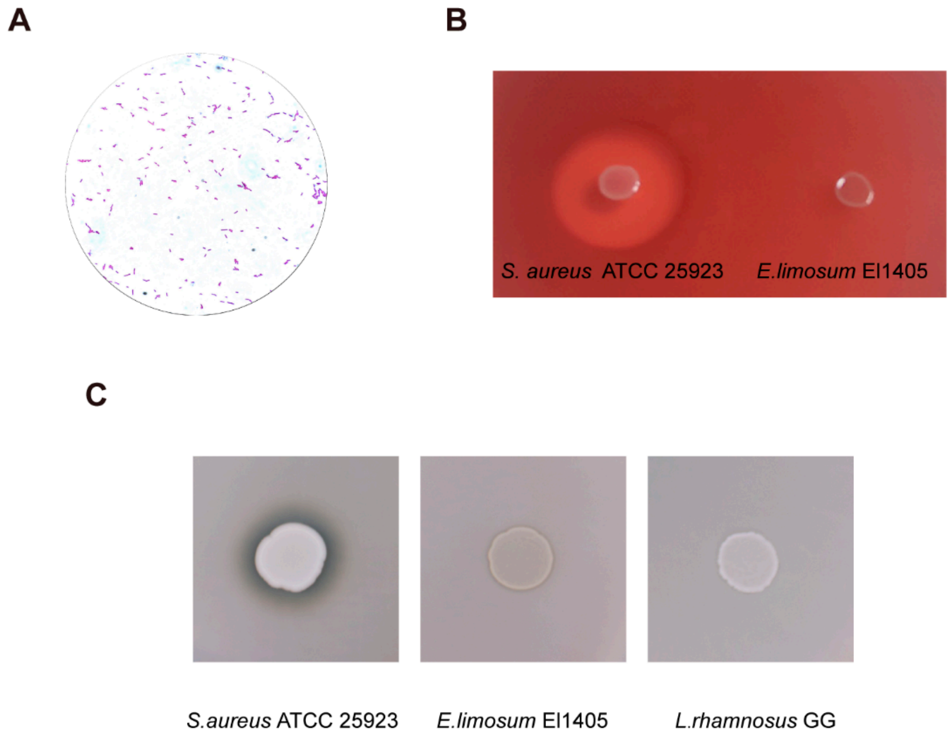

Figure S1. The results of *E. limosum* El1405 *in vitro*. (A) Gram staining of *E. limosum* El1405; (B) Hemolysis picture of *E. limosum* El1405; (C) Gelatinase activity of *E. limosum* El1405.

Supplementary Table S1. Antibiotic susceptibility of El1405.

classify	antibiotics	Sensitivity[1]	
		El1405	QC[2]
penicillins	penicillin	R	R
	ampicillin	S	R
	amoxicillin	S	S
carbapenems	imipenem	S	S
	meropenem	S	S
lincosamides	clindamycin	S	S
Cephalosporins	ceftriaxone	S	R
fluoroquinolones	moxifloxacin	S	S
Tetracyclines	tetracycline	S	S
nitroimidazoles	metronidazole	S	S

[1] S, susceptible; R, resistant. [2] QC, *Bacteroides fragilis* ATCC 25285.

Supplementary Table S2. The zone of inhibition diameter of El1405 live bacteria and supernatants.

	Live bacteria		Supernatant	
Pathogen	Inhibition Zone[1] (mm)	*p*-values[2]	Inhibition Zone[1] (mm)	*p*-values[2]
Negative control	7.59 ± 0.05		7.55 ± 0.19	
L. monocytogenes EGDe	11.78 ± 0.43	<0.0001	7.69 ± 0.11	ns
S. aureus ATCC 25923	41.55 ± 1.19	<0.0001	18.78 ± 0.55	<0.0001
S. Typhimurium SL1344	13.96 ± 0.38	<0.0001	18.36 ± 0.36	<0.0001
E. coli EDL933	13.63 ± 0.56	<0.0001	12.09 ± 0.67	<0.0001

[1] The results represent the mean ± SEM, with n = 3. [2] Differences among multiple groups compared to the negative control were evaluated using one-way ANOVA, followed by Dunnett's multiple comparison test, and ns means no significant difference.

Supplementary Table S3. Food and water intake of the negative control (NC) group and the experimental group.

Time	Food and water intake per mouse	Female				Male			
		NC	High	Medium	Low	NC	High	Medium	Low
Week 1	Water intake (mL)	3.48	3.62	3.50	3.62	4.19	4.29	4.29	4.48
	Food intake (g)	2.52	2.25	2.30	2.39	3.62	3.35	3.33	3.54
Week 2	Water intake (mL)	3.19	3.24	2.90	3.17	3.93	3.52	3.64	3.90
	Food intake (g)	2.08	2.10	2.07	2.16	3.44	2.94	2.94	3.44
Week 3	Water intake (mL)	4.05	3.57	3.29	3.52	4.76	4.29	4.12	4.38
	Food intake (g)	2.24	2.26	2.24	2.51	3.78	3.32	3.20	3.36
Week 4	Water intake (mL)	4.07	3.79	3.57	3.79	4.45	4.50	4.14	4.40
	Food intake (g)	2.58	2.56	2.39	2.68	3.69	3.46	3.41	3.65

No statistically significant differences were observed between all experimental groups and the control group ($p > 0.05$).

Supplementary Table S4. Hematological parameters in mice of the negative control group (NC) and the experimental group.

Parameters	Unit	Female (n=5)				Male (n=5)			
		NC	High dose	Medium dose	Low dose	NC	High dose	Medium dose	Low dose
WBC	10^9/L	4.04 ± 0.90	3.86 ± 1.20	3.30 ± 1.12	4.00 ± 0.30	3.94 ± 1.57	3.60 ± 0.38	3.82 ± 1.14	3.82 ± 1.02
Lymph	10^9/L	2.88 ± 0.74	2.92 ± 0.99	2.56 ± 0.87	2.92 ± 0.33	3.04 ± 0.99	3.06 ± 0.25	3.04 ± 1.01	2.86 ± 0.78
Mon	10^9/L	0.14 ± 0.05	0.12 ± 0.04	0.12 ± 0.04	0.14 ± 0.05	0.12 ± 0.11	0.10 ± 0.00	0.10 ± 0.00	0.16 ± 0.05
Gran	10^9/L	0.94 ± 0.33	0.82 ± 0.22	0.64 ± 0.21	0.92 ± 0.15	0.78 ± 0.48	0.80 ± 0.29	0.88 ± 0.22	0.90 ± 0.23
Lymph	%	73.38 ± 6.80	72.74 ± 3.46	75.66 ± 2.95	73.48 ± 3.62	78.16 ± 5.21	76.24 ± 6.01	77.42 ± 4.68	76.40 ± 8.13
Mon	%	4.04 ± 0.87	3.80 ± 0.70	3.74 ± 0.85	4.12 ± 0.69	2.64 ± 1.06	2.86 ± 0.69	2.60 ± 0.22	3.10 ± 1.15
Gran	%	23.16 ± 6.36	21.82 ± 2.93	20.60 ± 2.67	22.40 ± 3.00	18.30 ± 5.10	17.54 ± 5.62	17.76 ± 4.39	20.50 ± 7.18
RBC	10^{12}/L	10.71 ± 0.32	10.76 ± 0.55	10.59 ± 0.37	10.58 ± 0.28	10.57 ± 0.58	10.61 ± 0.18	10.95 ± 0.90	10.91 ± 1.16
HGB	g/L	149.60 ± 4.51	145.40 ± 4.34	149.00 ± 7.18	148.00 ± 6.36	157.00 ± 6.33	154.20 ± 6.94	162.20 ± 17.92	158.80 ± 15.87
HCT	%	57.58 ± 2.49	55.84 ± 2.52	55.88 ± 2.59	57.12 ± 3.16	54.86 ± 4.41	55.26 ± 1.76	54.64 ± 7.26	55.52 ± 6.63

MCV	fL	53.38 ± 0.98	53.60 ± 0.59	54.06 ± 0.23	53.68 ± 1.56	51.90 ± 1.67	51.72 ± 0.74	51.26 ± 2.46	51.34 ± 0.22
MCH	pg	13.86 ± 0.25	13.88 ± 0.13	13.94 ± 0.17	13.94 ± 0.34	14.84 ± 0.52	14.10 ± 0.21	14.64 ± 0.74	14.64 ± 0.34
MCHC	g/L	262.00 ± 6.44	260.00 ± 4.30	259.80 ± 3.70	258.60 ± 6.47	286.60 ± 15.24	272.00 ± 6.21	286.80 ± 20.61	281.80 ± 7.09
RDW	%	13.74 ± 0.72	14.20 ± 0.48	14.12 ± 0.53	14.36 ± 0.49	15.20 ± 1.18	14.16 ± 0.45	16.18 ± 2.64	15.24 ± 1.01
PLT	10^9/L	1058.00 ± 140.60	1101.00 ± 131.8	1080.00 ± 109.10	1051.00 ± 121.60	1523.00 ± 599.00	1179.00 ± 135.70	1929.00 ± 874.70	1476.00 ± 576.50
MPV	fL	6.62 ± 0.31	6.42 ± 0.13	6.48 ± 0.30	6.72 ± 0.19	7.16 ± 0.57	7.30 ± 0.16	7.30 ± 0.30	7.44 ± 0.47
PDW	%	17.14 ± 0.18	16.98 ± 0.13	17.04 ± 0.19	17.16 ± 0.21	17.42 ± 0.44	17.32 ± 0.23	16.98 ± 0.47	17.18 ± 0.29

WBC, white blood cell counts; Lymph, lymphocytes; Mon, monocytes; Gran, neutrophils; Lymph%, lymphocyte percentage; Mon%, monocyte percentage; Gran%, neutrophil percentage; RBC, red blood cell counts; HGB, hemoglobin; HCT, hematocrit; MCV, mean Corpuscular Volume; MCH, mean Corpuscular hemoglobin; MCHC, mean corpuscular hemoglobin concentration; RDW, red blood cell distribution width; PLT, platelet counts; MPV, mean platelet volume; PDW, platelet distribution width. All data are expressed as the mean ± SD (n=5). The hematological parameters of the experimental group were compared with those of the NC group, revealing no significant differences ($p > 0.05$).

Supplementary Table S5. Serum biochemical indicators of the negative control group (NC) and the experimental group.

Parameters	Female				Male			
(Unit)	NC	High dose	Medium dose	Low dose	NC dose	High dose	Medium dose	Low dose
ALT (U/L)	32.22 ± 2.97	32.02 ± 2.35	32.22 ± 2.46	32.60 ± 1.38	36.82 ± 3.87	38.74 ± 2.64	41.60 ± 7.21	37.68 ± 4.81
AST (U/L)	160.45 ± 17.87	157.77 ± 11.38	151.68 ± 10.28	161.35 ± 12.95	140.76 ± 7.33	165.62 ± 30.54	168.86 ± 30.48	153.98 ± 49.15
BUN (mmol/L)	13.72 ± 0.43	14.39 ± 0.58	13.90 ± 0.49	14.42 ± 0.74	12.62 ± 0.51	12.32 ± 0.49	13.36 ± 0.92	12.39 ± 1.77
CRE (µmol/L)	17.28 ± 0.53	17.60 ± 0.98	17.60 ± 0.32	17.80 ± 1.06	15.46 ± 0.94	15.58 ± 0.55	16.22 ± 1.30	15.02 ± 0.90
T-CHO (mmol/L)	2.71 ± 0.30	2.55 ± 0.21	2.61 ± 0.15	2.78 ± 0.19	3.96 ± 0.08	4.12 ± 0.30	3.97 ± 0.24	3.91 ± 0.21
TG (mmol/L)	0.27 ± 0.05	0.34 ± 0.06	0.32 ± 0.07	0.36 ± 0.11	0.54 ± 0.19	0.56 ± 0.13	0.46 ± 0.10	0.50 ± 0.09
CK (U/L)	483.38 ± 102.91	448.20 ± 97.89	434.54 ± 88.42	551.92 ± 91.40	777.20 ± 265.96	1006.42 ± 411.46	981.60 ± 414.29	612.26 ± 110.15
LDH (U/L)	2644.76 ± 142.35	2643.08 ± 132.74	2646.02 ± 96.59	2543.58 ± 190.28	1743.36 ± 283.24	1685.66 ± 528.25	2049.76 ± 344.81	1896.58 ± 371.80

ALT, alanine aminotransferase; AST, aspartate transaminase; GLU, glutamic acid; BUN, blood urea nitrogen; CRE, creatinine; T-CHO, total cholesterol; TG, triglyceride; CK. creatine kinase; LDH, lactate dehydrogenase. All data are expressed as the mean ± SD (n=5). There was no significant difference between the blood biochemical indexes of the experimental group and the NC group ($p > 0.05$).
